# Viral and cellular factors underlying neuropathogenesis in HIV associated neurocognitive disorders (HAND)

**DOI:** 10.1186/1742-6405-11-13

**Published:** 2014-05-19

**Authors:** Vasudev R Rao, Arthur P Ruiz, Vinayaka R Prasad

**Affiliations:** 1 Department of Microbiology and Immunology, Albert Einstein College of Medicine, 1300 Morris Park Avenue, Bronx, NY 10461, USA

## Abstract

As the HIV-1 epidemic enters its fourth decade, HIV-1 associated neurological disorders (HAND) continue to be a major concern in the infected population, despite the widespread use of anti-retroviral therapy. Advancing age and increased life expectancy of the HIV-1 infected population have been shown to increase the risk of cognitive dysfunction. Over the past 10 years, there has been a significant progress in our understanding of the mechanisms and the risk factors involved in the development of HAND. Key events that lead up to neuronal damage in HIV-1 infected individuals can be categorized based on the interaction of HIV-1 with the various cell types, including but not limited to macrophages, brain endothelial cells, microglia, astrocytes and the neurons. This review attempts to decipher these interactions, beginning with HIV-1 infection of macrophages and ultimately resulting in the release of neurotoxic viral and host products. These include: interaction with endothelial cells, resulting in the impairment of the blood brain barrier; interaction with the astrocytes, leading to metabolic and neurotransmitter imbalance; interactions with resident immune cells in the brain, leading to release of toxic cytokines and chemokines. We also review the mechanisms underlying neuronal damage caused by the factors mentioned above. We have attempted to bring together recent findings in these areas to help appreciate the viral and host factors that bring about neurological dysfunction. In addition, we review host factors and viral genotypic differences that affect phenotypic pathological outcomes, as well as recent advances in treatment options to specifically address the neurotoxic mechanisms in play.

## Introduction

HIV associated neurocognitive disorders (HAND) are a potential consequence of HIV-1 infection, and about half of all adults with AIDS suffer from neurological complications related to HIV-1 [1]. HIV-1 infection plays a pivotal role in HAND by generating products that lead to neurological damage in the central nervous system (CNS). HAND includes a spectrum of neurological disorders ranging from asymptomatic neurocognitive impairment (ANI), an intermediate form termed mild neurocognitive disorder (MND) and the severe form, HIV associated dementia (HAD) [2]. The relationship between the prevalence of these forms to each other and their occurrence in the overall population of HIV-1-infected individuals can be depicted in a pyramidal form, with the total number of HIV-1-infected individuals forming the base, with the upper layers having progressively fewer numbers of patients exhibiting ANI, MND and HAD. As the availability of highly active anti-retroviral therapy (HAART) has become more widespread worldwide, HIV-1-infected individuals are living longer. Although the incidence and prevalence of HAD have been reduced in the era of HAART, the prevalence of HAND overall is actually increasing worldwide. The increased lifespan of treated patients results in a chronic exposure of the brain to HIV-1 virions and viral proteins leading to inflammation, as well as a concomitant chronic inflammation in the peripheral immune system, leading to the accumulation of neurological damage [3]. The success of HAART in controlling peripheral viral load is not necessarily accompanied by reduction in the immune activation in the brain [4].

## Overview of HIV neuropathogenesis

Before embarking on delineating the viral and host factors that play central roles in HAND, it is helpful to have an understanding of the current working model for a mechanistic basis of HIV neuropathogenesis. Infiltration of monocytes into the brain is a hallmark of HAND [5]. Monocyte-derived macrophages (MDM) are one of the major types of cell that are infected by HIV-1 (CD4^+^ T lymphocytes and dendritic cells being the other two cell types). Once the HIV-1-infected macrophages have established residence in the CNS, they secrete chemokines that establish a chemotaxis gradient across the blood–brain barrier (BBB), which recruits more monocytic cells from the peripheral compartment into the CNS [6]. This influx of HIV-1-infected monocytic cells leads to the infection of other CNS-resident monocytic cells, namely perivascular macrophages and microglia [7], which in turn results in greater BBB damage and accelerates the rate at which HIV-1-infected and uninfected monocytic cells can enter the brain. Neurons do not support HIV-1-infection or replication, but they do express several cell-surface receptors (CCR5, CXCR4, NMDAR etc.) that render them sensitive to insults by viral proteins (e.g. Tat, gp120), inflammatory cytokines (e.g. TNF-α, IL1β) and small metabolites (e.g. nitric oxide, arachidonic acid, etc.) secreted by immune cells in the brain [8]. The key events that contribute to HAND include direct neuronal apoptosis, dysregulation of key neuronal support cells, and the loss of dendritic arbor.

## HIV-1 infection of Macrophages

The process of HIV-1 infection begins by HIV-1 binding to CD4 receptor on the target cell surface, through the viral envelope protein gp120 [9]. This binding induces a conformational change in gp120 that exposes a CD4-binding induced (CD4i) co-receptor binding site, which subsequently binds to either CCR5 or CXCR4 coreceptor [10]. The binding of gp120 to a co-receptor then exposes the fusion peptide in viral protein gp41, which is inserted into the cell membrane and drives fusion of the viral and cell membranes [11]. The co-receptor preference, which is determined primarily by the V3 loop in gp120 [12], designates a given HIV-1 isolate as either an R5 or X4 virus depending upon whether it uses CCR5 or CXCR4 coreceptor. Based on the fact that CCR5Δ32 mutation confers resistance to HIV-1 transmission [13], the field has come to the conclusion that only R5 viruses are transmissible and are the type of viruses detected systemically early in infection. However, later as the disease progresses, the predominant tropism switches to CXCR4-usage [14], at least in some of the cases (approximately 50%). CXCR4 usage is associated with faster viral replication and a more rapid CD4^+^ T-cell decline [14]. However, this switch is not obligatory for disease progression, as exceptions are observed in some parts of the world where non-B subtypes of HIV-1 are prevalent [15].

HIV-1 can productively infect both CD4^+^ T lymphocytes and monocytic cells/macrophages. Infection of monocytic/macrophage cells is primarily by M-tropic viruses driving fusion through the CCR5 coreceptor, but infection by T-tropic viruses through the CXCR4 coreceptor has also been observed [16]. While dendritic cells can also be infected by HIV-1, they do not support robust HIV-1 replication. However, they do play a crucial role in the systemic spread of HIV-1 due to their strategic presence in lymph nodes [17]. HIV-infection leads to a progressive decline in the population of CD4^+^ T cells, which is due to loss of both infected CD4^+^ T cells and uninfected cells (bystander killing) [18]. However, there is no systemic loss of monocytic cells, either infected or uninfected. It has been reported that monocytic/macrophage-tropic viruses, in addition to bearing gp120 that is able to recognize and bind CCR5 coreceptor, have evolved the capability to infect cells with a lower density of CD4 receptors on the cell surface and a greater fusion activity [19]. Furthermore, macrophages are also characterized by the budding of virions into internal multivesicular bodies, which are vacuoles within the cells, rather than budding through the plasma membrane directly to the external medium. This mechanism allows HIV-1 to ‘hide’ inside the infected macrophages, making macrophages one of the latent reservoirs of HIV-1 [20].

The predominant route of CNS exposure to HIV-1 is through peripheral blood monocytic cells/macrophages that have been infected by virus and transmigrate across the blood–brain barrier (BBB) [5]. Peripheral blood monocytes are categorized into classical “resting” monocytes that express only CD14 (CD14^++^CD16^-^), intermediate monocytes that maintain high levels of CD14, while gaining CD16 positivity (CD14^++^ CD16^+^), and non-classical “activated” monocytes that express high CD16 levels and lower levels of CD14 (CD14^+^CD16^++^) [21]. The CD16^++^ subset, while comprising only about 5-10% of the total circulating monocyte population, is overrepresented in the brains of patients with HIV encephalopathy [22,23]. CD16^++^ monocytes are more susceptible to HIV infection and support higher levels of viral replication [24]. Additionally, infected CD16^++^ monocytes have been shown to transmigrate more efficiently across the BBB in response to a chemokine gradient [25]. While CD4^+^ T lymphocytes have been suggested to play a role in trafficking HIV into the CNS, the vast majority of HIV-1 isolates derived from CNS tissue appear to be of macrophage-tropic lineage [26].

## CNS invasion: disruption of the blood brain barrier

The BBB is located at all points of interaction between the brain tissue and blood vessels and consists of astrocytic end feet and human brain microvascular endothelial cells (HBMEC). In addition to the barrier created by HBMECs and astrocytes, pericytes envelope the abluminal side of endothelium and increase the selectivity of the barrier [27]. Pericyte density in the brain is much higher than that observed in other organs, thereby constituting an additional barrier [28]. The BBB maintains the sanctity of the brain’s internal environment and provides an important physiologic and immunologic separation between the CNS and the peripheral circulation. HIV-1 infiltrates the BBB early after infection [29] using HIV-infected monocytic cells as a “Trojan horse” for entry into brain [30]. While low numbers of monocytes routinely enter the CNS as part of “immune surveillance” to replenish the population of resident macrophages, infection by HIV-1 increases the adherence of monocytes to endothelial cells, increasing their transmigration across the BBB [31]. The HIV-infected macrophages in the vicinity of the BBB secrete inflammatory cytokines and viral products that damage HBMECs, induce cellular oxidative stress and they also produce chemokines like CCL2 [32] that facilitate the build-up of additional immune cells near the barrier. The interplay between the secreted cytokines, viral products and HBMECs activates endothelial cells and increases surface expression of adhesion molecules on endothelial cells – which in turn accelerates the transmigration of infected macrophages across the BBB.

**Figure 1:**
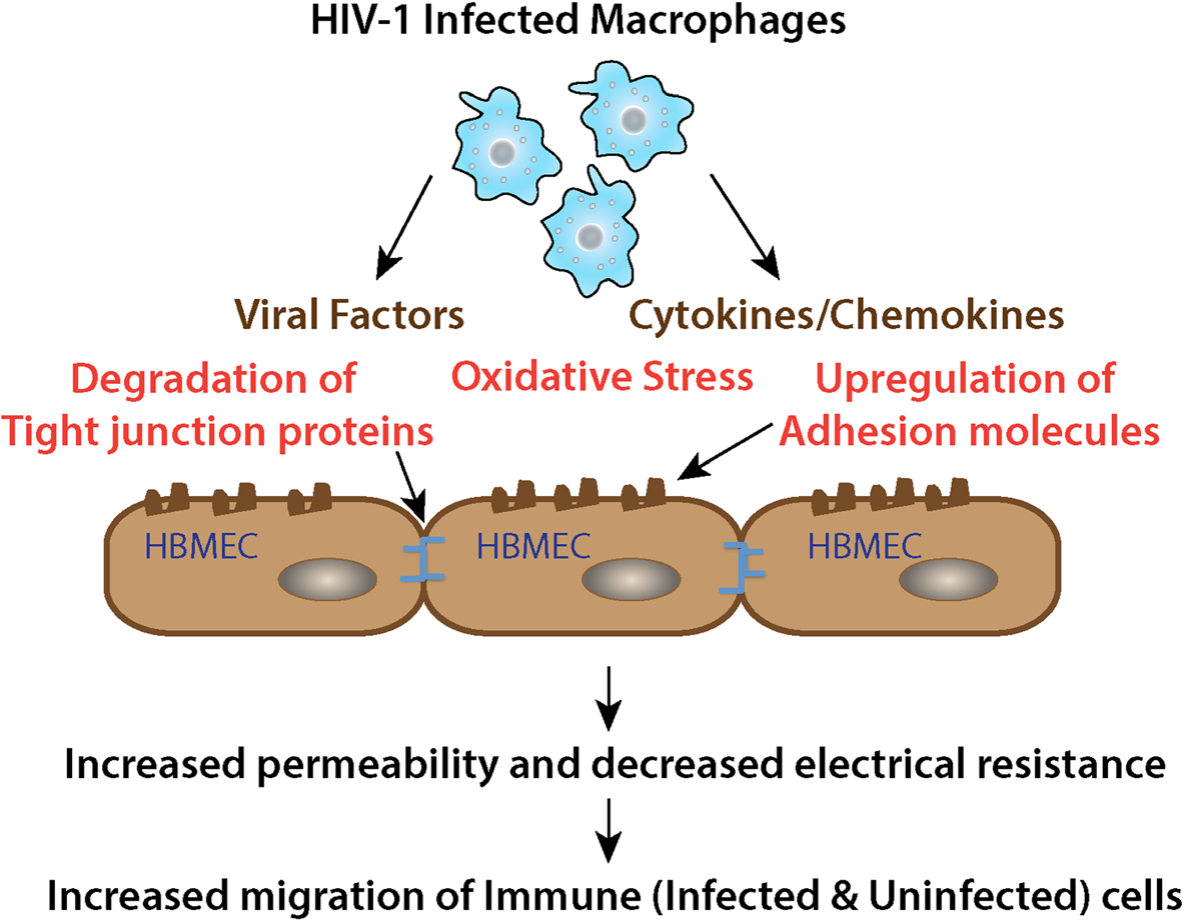
**Invasion of the blood brain barrier.** HIV-1 infected immune cells release toxic viral products and inflammatory cytokines leading to degradation of tight junction proteins, oxidative stress and up-regulation of adhesion molecules. This results in the increased permeability of the blood brain barrier and increased migration of immune cells across the barrier. HIV-1 infiltrates the BBB early after infection [29] using HIV-infected monocytic cells as a “Trojan horse” for entry into brain [30].

HIV-1 proteins Tat and gp120 both directly damage the BBB (Figure 1). Tat is a non-structural viral protein secreted by infected cells [33]. Tat mRNA [34], Tat protein [35] and antibodies to Tat [36] are detectable in the CNS of HIV-infected patients. Brain endothelial cells, when exposed to HIV-1 Tat, have been shown to alter the expression and distribution of tight junction proteins (claudins & occludins) [37]. Tat treatment of HBMECs *in vitro* up-regulates matrix metallopeptidase-9 (MMP-9), which in turn increases the permeability of brain endothelial cells by degrading occludins [38]. *In vitro* two-chamber models with HBMECs and astrocytes have also shown HIV-1 Tat causes dysregulation of nitric oxide production in brain endothelial cells [39] and induces secretion of CCL2, a key chemokine responsible for migration of immune cells [40].

Even though brain endothelial cells that line the BBB are not productively infected, several *in vivo* and *in vitro* studies have demonstrated that envelope glycoprotein gp120 causes a functional impairment at the BBB. The gp120 protein is found on the surface of virions and on infected cells as a homo-trimer, but gp120 is also shed from infected cells and found in soluble form as a monomer [41]. Studies involving HIV-1 gp120 transgenic mice, in which gp120 was expressed in thymocytes and the CNS, revealed that the permeability of the brain capillaries in mice is severely impaired [42]. Kanmogne *et al.* demonstrated, using a two-chamber BBB model, that the exposure of HBMECs to recombinant HIV-1 gp120 protein (from either X4 and R5 HIV-1 isolates) led to activation of proinflammatory and interferon-inducible genes, increased leukocyte adhesion, decreased trans-endothelial electrical resistance (TEER) and increased migration of monocytes across the barrier [43,44]. Removal of gp120 resulted in complete restoration of TEER and significant improvement in permeability. Exposure of HBMECs to gp120 *in vitro* resulted in increased expression of adhesion molecules (ICAM and VCAM), damage to the barrier and a consequent increase in monocyte migration across the monolayer of endothelial cells [45]. There was a dose dependent increase in cytotoxicity of HBMECs when exposed to recombinant gp120, and gp120-mediated HBMEC cytotoxicity has been shown to involve the p38 mitogen-activated protein (MAP) kinase pathway [46].

Thus, both HIV-1 proteins gp120 and HIV-1 Tat have been implicated in perturbing the blood–brain barrier. Delineating the precise roles of additional viral and host factors that directly perturb the BBB as well as identifying reliable biomarkers of BBB damage would be crucial to furthering our understanding of the mechanisms driving the pathology specific to the BBB in HAND. Such knowledge will accelerate the development of innovative treatment strategies, specifically aimed at preventing damage to the BBB and reducing the CNS invasion of infected macrophages.

## Mechanisms of neuropathogenesis

Although neurons cannot be infected by HIV-1, neuronal damage is a key feature in the development of HAND [47] Neurons are vulnerable to direct damage by several viral proteins. This vulnerability is mainly mediated through the presence of several neuronal cell surface receptors: the *N-methyl-D-Aspartate* receptors (NMDAR), the low-density lipoprotein receptor related protein (LRP), chemokine receptors CCR5 and CXCR4, and the dopamine transporter. Glutamate is the most abundant neurotransmitter in the brain, and it is responsible for propagating excitatory signal transmission by binding to NMDAR and opening cation specific channels in the cell membrane, allowing ingress of sodium and calcium ions and the egress of potassium ions [48]. LRP is a receptor involved in cholesterol trafficking in neurons, and also plays a role in signal transmission and inhibition of neuronal apoptosis [49]. Dopamine receptors and transporters are less widespread than NMDAR, but are importantly found in areas of the brain that are prone to high levels of HIV-1 infection, such as the striatum and substantia nigra, which are involved in executive function and the behavioral reward system [50], and there are lower expression levels of dopamine transporter in the striata of HIV patients with cognitive deficits [51].

**Figure 2:**
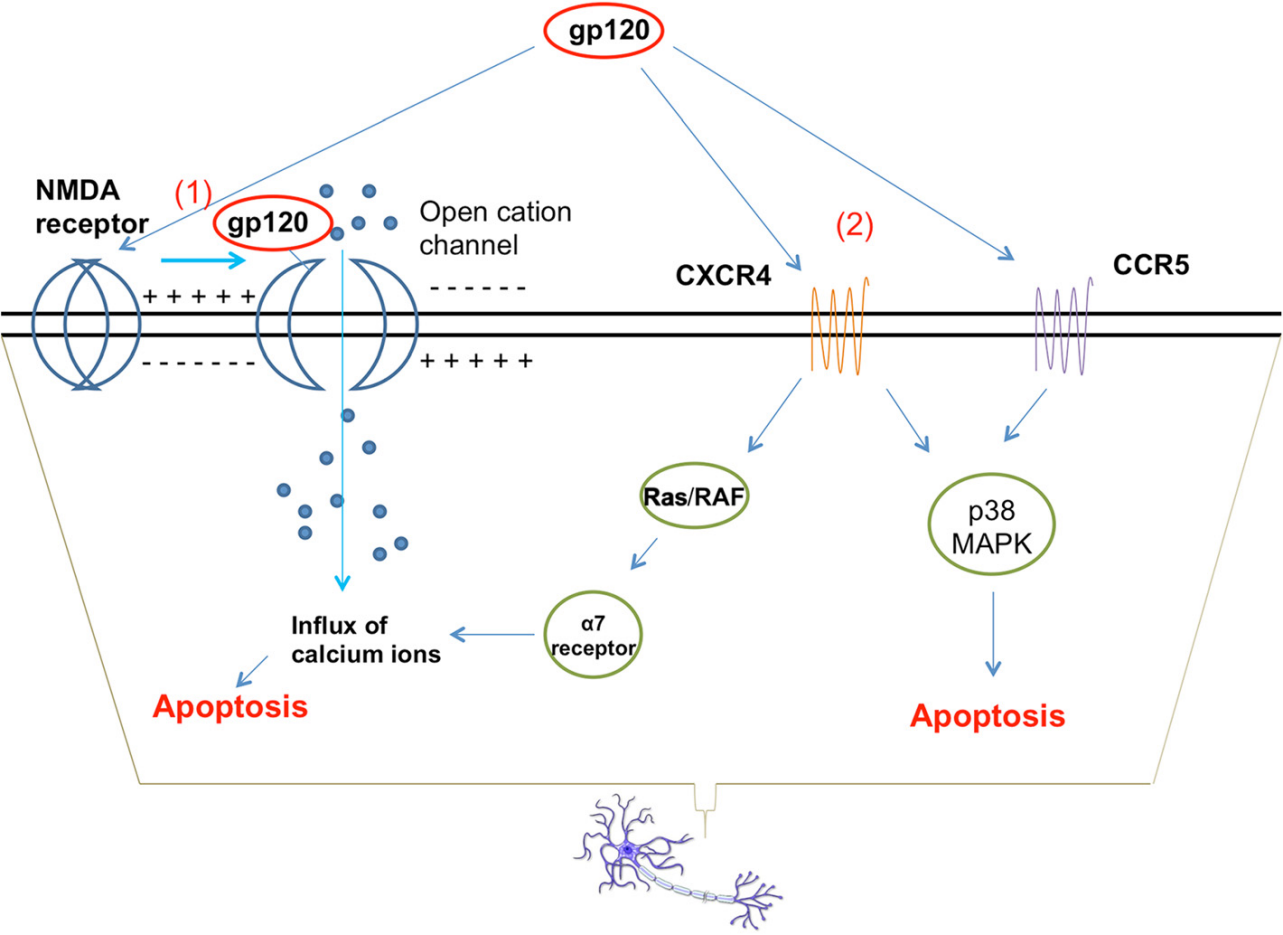
**Mechanisms of gp120 neurotoxicity.** (1) gp120 can bind to the NMDA receptor and lead to excessive opening of NMDAR-gated cation channels, allowing the influx of calcium ions to toxic levels [54]. (2) gp120 can directly bind to either CCR5 or CXCR4, activating an p38-MAPK mediated signaling cascade that leads to neuronal apoptosis [56]. The gp120-CXCR4 binding also up-regulates the expression of the nicotinic receptor α7, which increases cellular permeability to [Ca^2+^] influx and contributes to cell death [61].

The two major viral proteins that interact with the above receptors to cause neuronal injury are gp120 and Tat. Both monomeric and oligomeric gp120 have neurotoxic capabilities [52], and transgenic mice expressing gp120 have a spectrum of neuronal and glial changes resembling abnormalities in brains of HIV-1-infected humans [53]. HIV-1 gp120 directly binds NMDAR on human embryonic neurons and can cause a lethal influx of calcium ions [54] (Figure 2). HIV-1 gp120 can bind to either CCR5 or CXCR4 and induce death in neuroblastoma cells [52,55] (Figure 2). This apoptosis apparently takes place through a p38-MAPK-mediated signaling cascade [56]. Cognitive testing of gp120 transgenic mice showed age-dependent deficits in open field activity and spatial reference memory tests [57]. The natural ligands of both CCR5 (eg. CCL5, CCL3) and CXCR4 (CXCL12) were found to be neuroprotective against gp120 neurotoxicity [52,55,58,59]. However, CXCL12 displays neurotoxicity [56] after the N-terminal cleavage of a tetrapeptide in CXCL12 by MMP-2 [60]. Another factor up-regulated by the interaction of gp120 with CXCR4 is the neuronal nicotinic receptor α7, which increases cellular permeability to [Ca^2+^] influx and contributes to cell death [61].

**Figure 3:**
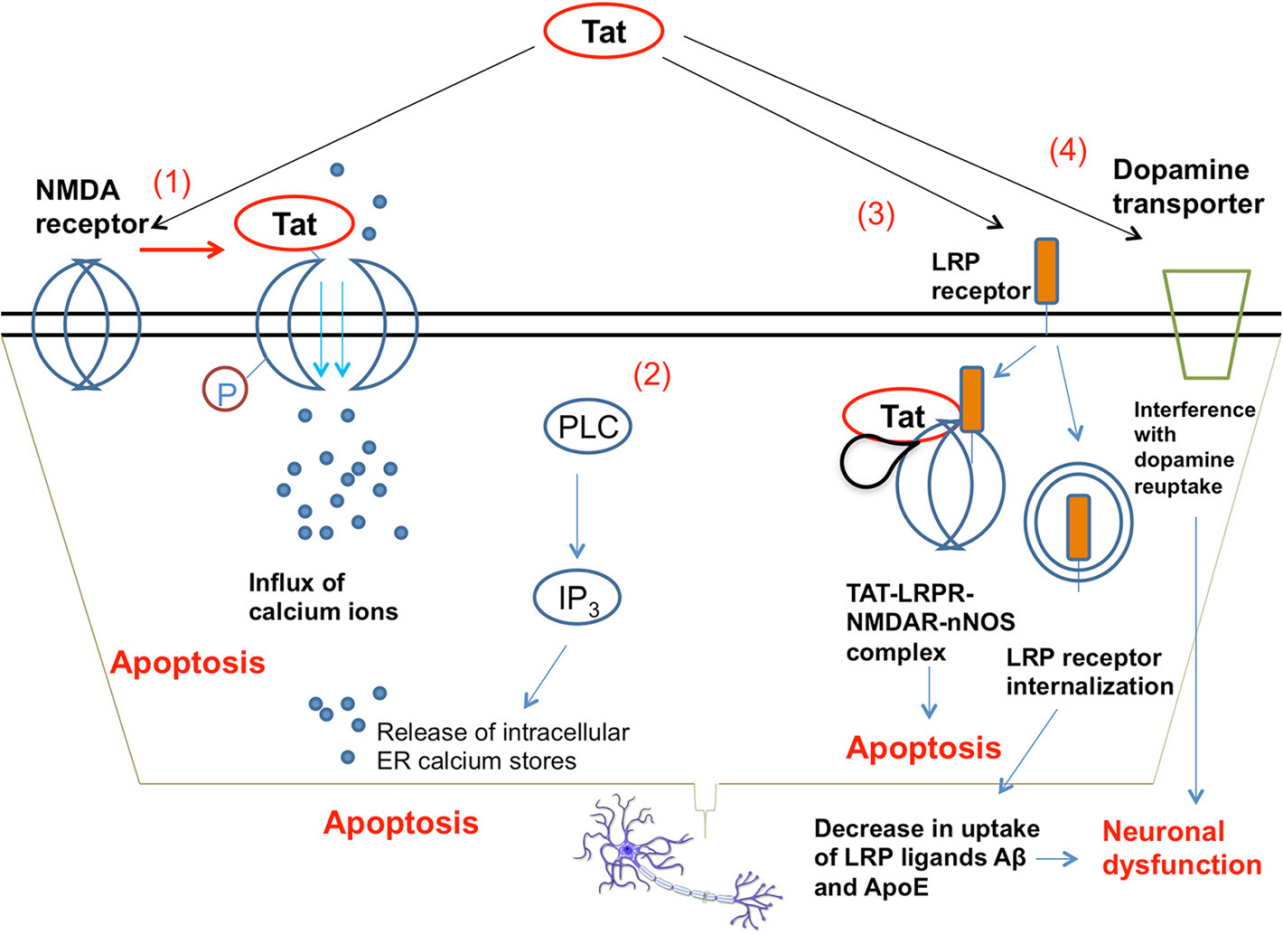
**Mechanisms of Tat neurotoxicity.** (1) Tat binds to the NMDA receptor and drives the phosphorylation of an intracellular NMDAR subunit, causing excess opening of cation channels and toxic accumulation of calcium [62]. (2) When applied to neurons, Tat is able to induce the activation of PLC and drive the IP3-mediated release of intracellular calcium from ER stores, further contributing to calcium toxicity and apoptosis [63]. (3) Tat can bind to LRP receptors, and be taken up as part of a macromolecular complex including NMDAR and neuronal nitric oxide synthase (nNOS) that induces cellular apoptosis [65]. Tat can also drive the internalization of the LRP receptor, reducing the uptake of LRP receptor ligands amyloid-β peptide and Apolipoprotein E, which may contribute to systemic neuronal dysfunction [64]. (4) Tat interferes with the activity of dopamine transporter, diminishing the reuptake of dopamine by pre-synaptic neurons and interfering with signal transmission [67].

The viral protein Tat also causes neurotoxicity via multiple pathways. Similar to gp120, Tat can activate NMDA receptors and drive the toxic influx of Ca^2+^ ions [62] (Figure 3). In addition to calcium dysregulation through the NMDAR, Tat can induce the phospholipase C-driven activation of inositol 1,4,5-triphosphate, which increases the intracellular levels of [Ca^2+^] by mobilizing stores in the endoplasmic reticulum and contributes to calcium toxicity and cell death [63]. Tat also binds LRP in neurons, causing LRP internalization and a decrease in uptake of natural LRP ligands such as amyloid-β peptide and Apolipoprotein E [64] (Figure 3). The interaction of Tat with LRP can lead to the formation of an apoptosis-promoting complex including postsynaptic density protein-95 (PSD-95), NMDA receptors and neuronal nitric oxide synthase (nNOS) [65]. Tat has been found to interfere with the expression of miRNAs in neurons, increasing the levels of CREB-targeting miR-34a and leading to neuronal dysfunction [66]. Tat can also interfere with the ability of dopamine transporter to reuptake dopamine [67]. This likely contributes to the particularly severe damage rendered to dopaminergic-rich regions in the brains of patients with severe HAND [51].

Other toxic viral proteins which have been found to activate caspases in neurons include Vpr [68] and Nef [69]. Vpu has been found to form cation-selective ion channels in a lipid bilayer membrane [70], though this effect has not been observed in neurons. Recently, stress pathways and accumulation of amyloid beta (Aβ) fibrils have been reported to be important in causing neuronal dysfunction. It has been suggested that an integrated stress response (ISR) pathway involving specific ISR proteins may underlie the neuroinflammatory processes observed in HAND [71]. It has also been reported that individuals with HIV encephalitis display a higher levels of intraneuronal Aβ accumulation in comparison with controls, suggesting that HIV impacts the clearance of Aβ in the brain [72].

Targeting the above discussed mechanisms of neuronal toxicity could yield therapies that can alleviate the symptoms and improve the quality of life for patients suffering from HAND.

## Macrophage and microglial activation

In addition to toxic viral proteins, HIV-1-infected macrophages and microglia release a variety of neurotoxic host factors that contribute to neuronal injury. These can be broadly categorized as proinflammatory cytokines, chemokines, and small molecules. The proinflammatory cytokines induced by HIV-1-infection of macrophages include TNF-α, IL-1β, IL-6, IL-8 and IFN-α. TNF-α has been shown to also inhibit glutamate uptake by astrocytes, leading to a extracellular buildup of glutamate that can lead to neuronal excitotoxicity [73]. TNF-α has also been found to have a neuroprotective role, through the TNFR2 mediated activation of NF-κB through a PI3 kinase [74], so its role is more complicated than simply that of a neurotoxin. IL-1β is released from macrophages in response to protein kinases induced by gp120 [75], and both IL-1β and TNF-α were found to dysregulate glutamate production in neurons through the induction of glutaminase [76]. IL-6 is also directly induced in macrophages by gp120, and leads to the formation of large cytoplasmic vacuoles in neurons that disrupt neuronal function [77]. Excess levels of IFN-α have been correlated with severity of HIV dementia [78]. Treatment of MDM with IFN-α (or HIV-1 infection) has been reported to increase glutaminase (GLS1) expression through phosphorylation of STAT1, which was shown to act through GLS1 promoter [79]. In addition to inflammatory cytokines, small molecules released by HIV-1-infected macrophages such as platelet activating factor (PAF) and quinolinic acid play a key role in neurotoxicity via NMDAR dysregulation [80,81].

Microglia are mesoderm-derived cells whose population is established in the brain perinatally. They are responsible for immune surveillance of the CNS, and are the primary source of inflammatory cytokines in the brain [82]. Infected microglia are thought to be the main source of giant multinucleated cells, which are a hallmark of HIV associated dementia and an important source of inflammatory cytokines in the CNS of HIV-1 patients [83]. Microglia are a major source of neuronal excitotoxicity, releasing glutamate in response to HIV-1 infection [84]. Even in patients who are able to effectively control HIV infection via ART, increased microglial activation has been shown to correlate with poorer executive performance [85]. Treatment of fetal human microglia with recombinant Tat induces the production of a number of chemokines, such as CCL2 and CCL5, involved in the development of HAND [86]. Dysregulated chemokines released from microglia have an especially potent impact on neurons, due to the presence of several chemokine receptors on neuronal cell surface, including CCR1, CCR4, CCR5, CCR9/10, CXCR2, CXCR4, and CX3CR1 [87].

## Astrocytes: no longer a passive bystander

Over the past 10 years, a significant amount of research has gone into understanding the extensive role played by astrocytes in the CNS, overturning their previous status as merely space holders, structural scaffolding, and scavengers. Their newly described roles include uptake of neurotransmitters, modulation of synaptic transmission, BBB maintenance, vaso-modulation, and long-term potentiation. The new concept of neuronal-glial intercommunication, where astrocytes play a dynamic role by integrating neuronal inputs and modulating synaptic activity, has helped us better comprehend astrocyte dysfunction in the context of HAND [88,89].

It has been recently shown that astrocytes can be infected by HIV-1 *in vitro*, and additionally that infected astrocytes can impair BBB function [90], but the infected astrocytes are rarely seen in patient brain autopsies (although one recent study has shown 20% of the astrocytes are infected in patients with HAD [91]). Furthermore, recombinant Tat protein has been shown to be responsible for the induction of chemokines [92], cytokines [93] and nitric oxide synthetase [94] in cultured primary human astrocytes. For example, Tat has been shown to induce astrocytes to produce platelet-derived growth factor BB (PDGF-BB), which in turn induces the production of CCL2 [95]. HIV-1 Tat can also up-regulate the expression of MMP-9 via MAPK-NF-κB-dependent mechanism, and MMP-9, in turn, disrupts the BBB [96].

Exposure of astrocytes to gp120 causes up-regulation of IL-6 and TNF-α [97], and increases the release of glutamate and potassium, which leads to toxic increases in calcium in neurons and astrocytes [98]. Using Affymetrix microarray analysis, Wang *et al.* showed that primary human astrocytes, when exposed to HIV-1 or gp120 *in vitro*, have an impaired ability to transport L-glutamate, and the authors ascribed this defect to transcriptional inhibition of the EAAT2 glutamate transporter gene [99]. More recently, Fernandes *et al.* have used an animal model to show that gp120 prevents the uptake of extracellular glutamate by astrocytes, leading to a disruption of glutamate-glutamine homeostasis and a consequent impairment of memory [100]. Release of toxic cytokines, inability to take up excess glutamate and damage to the BBB make astrocytes a central offender in the pathogenesis of HAND.

## Neuroprotective factors

Macrophages, microglia and even neurons themselves produce a number of neuroprotective mediators of inflammation. Neurotoxicity seen in HAND is the net result of the effects of neurotoxic insults and the action of neuroprotective host factors. In the quest to define neurotoxic agents responsible for HAND, the neuroprotective processes often go unnoticed. Platelet derived growth factor (PDGF) confers neuroprotection against gp120 toxicity by stimulating the PI3K/Akt pathway [101], and the pretreatment of neuronal cells with PDGF-CC abrogated Tat-mediated neurotoxicity by mitigating apoptosis and neurite loss [102]. Astrocytes exposed to HIV-1 or chronically infected with HIV-1 express the tissue inhibitor of metalloproteinases-1 (TIMP-1), which is shown to have a neuroprotective role when primary human neurons are exposed to HIV-1 [103]. It has also been reported that exposure of astrocytes to HIV-1 gp120 induces the expression of nuclear factor erythroid-derived 2-related factor 2 (Nrf2), which plays a role in the stimulation of anti-oxidant defensive enzymes [104].

Fractalkine (FKN/CX3CL1) is a chemokine produced by neurons, and it plays an important role in communication with microglia, which abundantly expresses the FKN receptor CX3CR1 (neurons also express this receptor but less abundantly). FKN plays an important role in neuroprotection and helps reduce gp120 toxicity via activation of ERK1/2 and CREB (this effect was seen both in the presence and absence of co-cultivated glial cells) [105]. Increased levels of FKN were found in the neurons of patients with HIV encephalitis, and FKN was found to potently drive the migration of monocytes across an *in vitro* trans-well system, as well as inhibit the neurotoxic effects of Tat protein in rat cerebellar neurons [106]. Also the chemokine CCL3L1 protects against gp120 neurotoxicity by phosphorylation of CREB, which promotes the transcription of the cell-survival gene bcl-2 [107]. The routes of action of these host mediators against viral neurotoxins may offer potential avenues of therapeutic intervention that capitalize on existing pathways of neuroprotection.

## Host genetic determinants of neurotoxicity

Several important host gene polymorphisms have been associated with an effect on the transmission of HIV-1 and/or AIDS disease progression (e.g., CCR5-Δ32 [108], CCL31 [109]). Similarly, several host gene polymorphisms have been linked with effects on the susceptibility to neurocognitive impairment. A point mutation identified in CCR2 (V64I) that had been associated with slower immunosuppressive disease progression [110], has also been shown to be specifically associated with a slower progression of neurocognitive impairment [111]. This V64I variant was not associated with a difference in plasma or CSF viral load, suggesting that its impact could be mediated through a mis-regulation of the inflammatory response rather than through differences in viral replication. A polymorphism in the TNF-α gene increases its production in response to bacterial LPS, and this allele has been found in an increased frequency of patients with HAD [112]. An allele of CCL2, 2578G confers a 50% reduction in risk of acquiring HIV-1 infection. However, the same allele, subsequent to HIV infection, was also associated with a 4.5 fold increased risk of HAD [113]. This 2578G allele resulted in a greater transcriptional output of CCL2, which could exacerbate the development of HAND by increasing the influx of monocytic cells in response to CNS infection.

## HAND co-morbidity with drugs of abuse

Neurocognitive disease in HIV-patients can be greatly exacerbated by abuse of drugs such as opiates, methamphetamine and cocaine. Injection drug users (IDUs) are at particular risk, as injection drug use remains an important route of HIV-1 transmission [114]. In addition, HIV-patients addicted to drugs of abuse are less likely to adhere to antiretroviral drug regimens. Opioid abuse is associated with faster HIV-1 disease progression, including increased risks of neurocognitive problems. While the “typical” opioid abuser has previously been perceived as a heroin IDU, there is a growing problem with the rising numbers of people abusing prescription opiates – a 2008 report indicated that while 400,000 Americans used heroin within one year, an estimated 12 million Americans abused prescription opiates within the same timeframe [115]. The mechanisms of opioid-mediated injury in conjunction with HIV-CNS infection are well documented. μ-opiod receptor (MOR) is one of the primary routes of opiod signaling [116]. While morphine itself leads to the down-regulation of MOR in cultured microglia, treatment of microglia with morphine and Tat reverses this down-regulation, providing a potential feed-forward mechanism of higher numbers of MOR at the cell-surface available to propagate further morphine-Tat mediated neurotoxicity [117]. Doxycycline-inducible Tat transgenic mice (that express HIV-1 Tat in astrocytes) that were administered both doxycycline and morphine showed a marked decrease in the density of dendritic spines in the striatum, compared with mice given either doxycycline or morphine alone [118]. PDGF-BB was also able to protect human neurons from synergistic Tat/morphine damage by inhibiting production of ROS and caspase-3 through the activation of the PI3K pathways [119].

Morphine also acts synergistically with gp120 to promote neuronal death through p38 MAP kinase signaling [120]. Mice that were transgenic for gp120 and subjected to morphine withdrawal failed to normalize oxidative capacity compared with non-transgenic mice subjected to morphine withdrawal. The same study also found failure to recover from synaptic damage in gp120-transgenic mice experiencing withdrawal compared with non-transgenic mice [121]. Tat-induced release of CCL2 from astrocytes is further enhanced by morphine, and can increase the chemotactic migration of a microglial cell line towards affected astrocytes [122]. HIV-1 patients using opiates were found to have higher levels of microglial activation in their brains [123]. There is also evidence of opioid use having an effect on HIV-1 infection and replication: morphine significantly enhanced the infectivity of human macrophages, possibly by an increase in CCR5 expression [124]. A more recent study showed that treatment of monocytes with morphine down-regulated several anti-HIV-1 microRNAs, which rendered the monocytes more susceptible to infection [125]. Administration of morphine to rhesus macaques before infection with SIV resulted in a higher viral set point compared with a non-morphine control group [126].

Methamphetamine (METH) use has been found to exacerbate brain injury and neuronal loss in HIV-1 patients, particularly in populations of interneurons in dopamingeric areas (DA) where the drug acts on the behavioral reward system [127]. METH potentiates a Tat-mediated decrease in dopaminergic release in the cultured human fetal neurons [128]. In the human neuroblastoma cell line SH-SY5Y, treatment with both METH and Tat produced significantly higher levels of both apoptosis and formation of autophagosomes than either factor by itself [129]. In cultured human astrocytes, METH was found to potentiate the gp120-mediated release of the pro-inflammatory cytokine IL-6, mediated through the PI3K/Akt and NF-κB pathways [130]. Treatment of METH on astroglioma cells transfected with a Tat expression construct exacerbated Tat-induced down-regulation of the neuroprotective factor β-catenin [131]. Furthermore, HIV-patients using METH were found to have significantly higher viral loads than non-METH users. This was found to be a result of the interference of METH with antiretroviral therapy [132]. METH also interferes with the normal antigen processing and phagocytic functions of macrophages [133], enhances the susceptibility of macrophages to HIV-1 infection by up-regulation of CCR5 [134] and increases HIV-1 replication in CD4 T cells and monocytes [135].

Cocaine is also known to accelerate the pathology of HAND. The drug binds the dopamine transporter and reduces the rate of dopamine uptake. Cocaine potentiates Tat-mediated neurotoxicity through the action at D1 receptors [136], Cocaine administration to SHIV-infected simian macrophages enhanced viral replication through increases in NF-κB signaling and IL-10 [137], and cultured human endothelial cells exposed to cocaine experienced an up-regulation in adhesion molecules, resulting in increased monocyte transmigration across the BBB [138].

Interestingly, according to recent studies cannabinoids (CB) appear to actually mediate a degree of neuroprotection in the context of CNS-HIV-1 damage. Synthetic CBs acting on the CB2 receptor (CB2-R) were able to mitigate gp120-induced damage in both human dopaminergic neurons and CB-2R expressing microglia [139], and a CB2 agonist was found to rescue deficits in neurogenesis seen in gp120-expressing transgenic mice [140].

## Viral genetic determinants

HIV-1 is phylogenetically divided into four groups: M, N, O and P, each representing a successful zoonotic event from primates – group M being the most widespread. Group M is further divided into subtypes A, B, C, D, F, G, H, J, K as well as a number of circulating recombinant forms (CRFs) of these subtypes. The global distribution of HIV-1 subtypes is region-specific, with a given geographical region correlating with the presence of a distinct subtype, a CRF or a particular combination of subtypes and CRFs [141]. Such a highly region-specific distribution is thought to be due either to a founder effect, to host-population genetics selecting for specific subtypes of HIV-1, or a combination of both [142]. Region-specific subtype-distribution has allowed researchers to correlate region-specific disease severity to subtype-specific viral gene polymorphisms, as documented in clinical studies. For example, reports from India, where HIV-1 subtype C is the predominant subtype, pointed to a lower incidence (0 - 4%) of severe forms of HAD [143,144], in contrast to geographic regions where subtype B HIV-1 is prevalent, which reported pre-HAART incidence levels of 15 - 30% [145,146].

**Figure 4:**
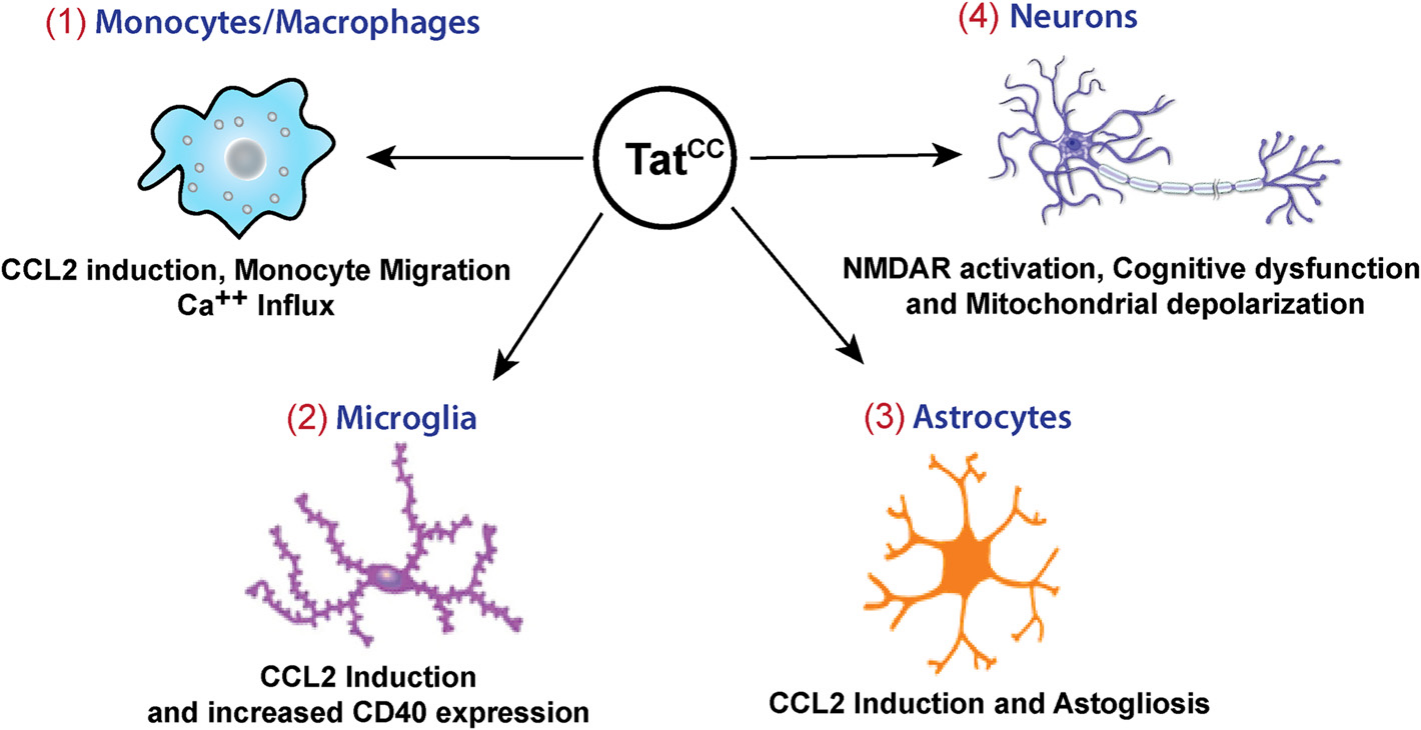
**Contribution of the dicysteine motif in Tat to neuropathogenesis.** (1) Tat is secreted by infected macrophages and microglia in the vincity of the blood brain barrier resulting in damage to the integrity of the barrier. Dicysteine Tat can act as a ligand on certain chemokine receptors expressed by circulating monocytes (i.e. CCR2, CCR3, CCR5), and promote monocyte migration across the BBB into the CNS [148]. Clade C Tat lacking the intact dicysteine motif fails to attract monocytes in a two chamber migration assay [149]. (2) Dicysteine Tat (TatB but not TatC) also induces higher expression of CCL2 and activation marker CD40 in microglial cells than non-dicysteine Tat [150]. (3) When applied to astrocytes, dicysteine Tat induces higher levels of CCL2 [149] and in HIVE SCID Mouse model astrogliosis is more pronounced in the presence of HIV-1 with an intact dicysteine motif [151]. (4) In neurons, it has been proposed that the Cys31 in the Tat dicysteine motif specifically disrupts the disulfide bond between Cys 744 and Cys 798 on the NR1 subunit of the NMDA receptor, leading to persistent activation of the NMDA receptor [152]. The increase in Tat-mediated apoptosis in dicysteine Tat is also seen in human primary neurons (mediated by increased levels of cleaved caspase-3), in addition to higher levels of ROS and increased levels of mitochondrial depolarization [149]. Primary Human neurons exposed to viral supernatants from HIV-1 with intact dicysteine motif exhibit neuronal apoptosis and HIV-1 SCID mice exposed to the same supernatants have cognitive dysfunction [151].

Our laboratory previously investigated whether genetic differences between the circulating isolates are responsible for the wide variation in incidence of HAD in different geographic regions. We reported that a majority (88%) of HIV-1 subtype C isolates worldwide have a Tat protein with a Cys31Ser polymorphism that disrupts a dicysteine motif, which is a key element of Tat protein’s homology to β-chemokines [147] (Figure 4). In contrast, the Tat proteins encoded by 99% of non-subtype C HIV-1 isolates maintained a C31 residue at that position. We demonstrated that Tat protein of subtype C HIV-1 isolates with a C31S polymorphism is defective for monocyte chemotaxis function in vitro [147]. As the infiltration of monocytic cells to the brain is a hallmark of HAD and plays a central role in triggering the inflammatory onslaught on neurons, we hypothesized that the C31S polymorphism in Tat protein of subtype C HIV-1 isolates contributes to the mechanistic basis for low incidence of HAD reported in India, as fewer monocytic cells would migrate to brain and result in little neuronal damage.

In subsequent studies, several other groups have showed that recombinant subtype C Tat protein has a decreased potential for direct neurotoxicity [149,152] and a decreased ability to induce inflammatory chemokine and cytokines [153-155]. In order to examine whether the C31S polymorphism is associated with biological differences in neuropathogenesis, we compared cognitive dysfunction and pathological changes induced by representative subtype C and B isolates in a SCID mouse HIV encephalitis (SCID-HIVE) model [155]. This allowed us to compare two genetically distinct HIV-1 isolates employing a single host genetic background. These studies revealed that subtype C isolates with a C31S polymorphism have lower potential to cause neuropathology and cognitive dysfunction [155] (Figure 4). In primary monocytes, subtype B Tat was found to induce higher amounts of pro-inflammatory factors than subtype C Tat, such as TNF, IL-6, CCL2, and IDO (an enzyme in the kynurenine pathway that leads to production of neurotoxic quinolinic acid) [153]. HIV-1 TatB has also been shown to disrupt the integrity of brain microvascular endothelial cells to a greater extent compared to Tat C [156]. Also, TatB, compared with TatC, can induce higher levels of IDO in human primary astrocytes [157], and TatB also induces higher levels of CCL2 in microglia [150] and primary astrocytes than TatC (this higher CCL2 induction was specifically associated with the presence of the dicysteine motif) [149] (Figure 4). Astroglioma cells that were either transfected with a Tat expression construct or treated with recombinant Tat showed a decrease in β-catenin, a transcriptional co-activator in the Wnt signaling pathway involved in neuroprotection. This β-catenin decrease was specific to the dicysteine motif in Tat, as neither TatC nor a TatB mutant with a disrupted dicysteine motif (C30G) were able to bring about this decrease [158].

The low incidence of HAD caused by subtype C HIV-1 in India was further corroborated by the absence of HAD in Ethiopia, as measured by neurocognitive tests using International HIV Dementia (IHD) scale. Subtype C HIV-1 is also widespread in the Southern African countries. Interestingly, however, recent cohort studies in South Africa and Botswana, where C is the predominant subtype, showed that the incidence of HAD is much higher than that in India at 25 and 38% respectively [159-162]. In light of our earlier findings indicating reduced neuropathogenicity for subtype C HIV-1 [155], we were intrigued by the increased incidence data reported for subtype C HIV-1 cohorts in Southern Africa. While it may be generally assumed that subtype C HIV-1 from different regions of the world are highly related, studies on the phylogenetic and molecular clock analysis of gp120 sequences of Indian HIV-1 isolates have shown that their origin in India occurred much later than that of South African subtype C HIV-1 [163,164]. Therefore, we initiated a multi-site study to investigate phylogenetic differences between Southern African (South Africa and Zambia) and South Asian (India and Bangladesh) subtype C HIV-1 isolates. Our results showed that, with respect to both the whole virus genome sequence as well as sequences encoding Tat protein, the Indian HIV-1C isolates are genetically distinct from those in Southern Africa. More importantly, HIV-1C isolates with an intact dicysteine motif in Tat protein (C31) were quite rare (2 - 3%) in India, while they were significantly more frequent in the Southern African countries (11 - 26%). Thus, the frequency of HIV-1C isolates with a C31 in Tat broadly paralleled the incidence of HAD in these countries. Neurocognitive tests in SCID-HIVE mice, using a representative subtype C from Southern Africa (HIV-1_1084i_) with an intact dicysteine motif in Tat (C31) and comparing it to subtype C from India (HIV-1_IndieC1_) lacking an intact dicysteine motif in Tat (C31S), showed that mice exposed to subtype C from Southern Africa exhibited significantly greater cognitive dysfunction compared to subtype C from India [151], especially when the testing paradigm presented a higher level of memory load. These findings lead to three main conclusions. One, intra-subtype variation exists and such variation can involve viral genes in HIV-1 that play a direct role in neurotoxicity. Two, it is more accurate to correlate differential disease outcomes in two geographical regions being compared, to differences in neurovirulence signatures rather than to differences in subtypes as a whole. Three, once further confirmed in a clinical study, it should be possible to perform a genetic screening in a subtype C HIV-1 epidemic for Tat genotype to gauge the risk of neurological dysfunction at the time of initial diagnosis.

Additional genetic determinants in Tat, other than C31, that play a crucial role in the pathogenesis of HAND are unknown, but are likely to exist. Further studies on differential neuropathogenesis potential of HIV-1 variants in different geographic regions will be informative. Similarly, considering the fact that gp120 causes neuropathogenesis via multiple pathways, it is also possible that there are genetic differences in gp120 between viral variants in their ability to cause HAND via gp120-mediated neurotoxicity. Identification of such variants and their impact on the pathogenesis of HAND will help dissect the various types of neurotoxic insults involving different cellular receptors and develop better therapeutics and/or diagnostics.

## Treatment modalities for HAND

HAART significantly lowers the viral load in both the periphery and the CNS, and has been crucial in significantly decreasing the incidence and prevalence of severe forms of HAND [165]. However, recent clinical studies and a meta-analysis [1,166,167] in patients on HAART revealed milder forms of neurological dysfunction (ANI & MND) in greater than half of HIV-positive patients. In studies examining individuals with ART-controlled HIV viremia, HIV DNA in PBMCs correlated with regional brain atrophy highlighting the contribution of peripheral viral reservoirs to CNS pathology [168]. Even the modified HAART regimens that include drugs with better CNS penetration effectiveness (CPE) scores, termed neuro-HAART, have had limited success among patients [169-171]. Researchers have suggested various approaches to targeting latent CNS HIV reservoirs and sources of neuroinflammation such as cytokines, chemokines and oxidative stress that are in play inside the CNS and are refractory to antiretrovirals. Use of anti-inflammatory and anti-excitotoxic drugs like Minocycline (second-generation tetracycline derivative) [172] and Memantine (voltage-dependent, open channel NMDAR blocker) [173], that can cross the BBB at substantial levels and that have been shown to be neuroprotective in SCID HIVE mice and SIV models, are currently being investigated as adjuvants to treat HAND. Several neuroprotective factors, both endogenous (as discussed above) and exogenous (e.g., pDING; [174]) are also being proposed as alternate treatments, with a focus towards eliminating neuroinflammation caused by exposure to HIV-1 [101-103]. Intra-nasal delivery of neurotropic protective factors such as insulin, IGF-1 and neurotrophin has been shown to mitigate many of the behavioral and pathological deficits in gp120 transgenic mice [175]. *In vitro* studies, using innovative delivery techniques such as gold and lipid based nano-particles, have been shown to be effective in delivering HAART and anti-inflammatory/anti-excitotoxic agents across the blood brain barrier [176] to specifically target CNS reservoirs. NanoART treatments in humanized mice reduced viral loads and protected CD4 T-cell populations [177]. Natalizumab, a monoclonal antibody against alpha-4-integrin known to block trafficking of leukocytes across the blood–brain barrier, has been shown to be effective in preventing HIV-1 infected cells from breaching the BBB in a SIV model by Campbell and colleagues [178]. These new and innovative treatments, in combination with Neuro-HAART, could be useful in treating HAND in the aging at-risk HIV-1 infected population. Thus, current approaches to intervention of HAND include not only the careful choice of antiretrovirals with better CNS penetration and efficacy, but also the future use of neuroprotective agents or the modulation of neuroprotective host factors.

## Conclusions

Our precise understanding of host factors that mediate the neurotoxic insult launched by exposure of CNS to HIV-1-infected cells, HIV-1 or specific HIV-1 proteins has increased in the recent past. In addition to increased knowledge about the pathways of neurotoxicity, we are also learning about the regional genetic variations in viral genes leading to intra-clade, region specific differences in disease severity. The discussion above clearly indicates that numerous opportunities exist to specifically block the neurotoxicity of viral and host proteins as well as potential neuroprotective factors that could be augmented to help alleviate the negative impact of HIV-1 on the brain.

## Abbreviations

CNS: Central nervous system; HAND: HIV-1 associated neurological disorders; ANI: Asymptomatic neurocognitive impairment; MND: Mild neurocognitive disorder; HAD: HIV-1 associated dementia; HAART: Highly active anti-retroviral therapy; ART: Anti-retroviral therapy; BBB: Blood-brain barrier; HBMEC: Human brain microvascular endothelial cells; MAP: Mitogen-activated protein; NMDAR: N-methyl-D-Aspartate receptors; LRPR: Lipoprotein receptor related protein receptors; PAF: Platelet activating factor; PDGF: Platelet derived growth factor; CRFs: Circulating recombinant forms; SCID-HIVE: SCID mouse HIV-1 encephalitis; CPE: CNS penetration effectiveness.

## Competing interests

The authors declare that they have no competing interests.

## Authors’ contributions

All three authors wrote sections of the manuscript. VRR developed the arrangement of the various sections and VRP was responsible for the integration of the sections. All authors read and approved the final manuscript.
